# Plant-Derived Foods and Medicines as Modulators of the Gut Microbiome: Molecular Interactions and Implications for Disease and Therapy

**DOI:** 10.3390/molecules31122191

**Published:** 2026-06-22

**Authors:** Gabriela Mitea, Verginica Schröder, Marius Daniel Radu, Horațiu Mireșan, Irina Mihaela Iancu

**Affiliations:** 1 Department of Pharmacology, Faculty of Pharmacy, Ovidius University of Constanta, 900470 Constanta, Romania; gabriela.mitea@365.univ-ovidius.ro; 2 Department of Cellular and Molecular Biology, Faculty of Pharmacy, Ovidius University of Constanta, 900470 Constanta, Romania; 3 Faculty of Natural and Agricultural Sciences, Ovidius University of Constanta, 900470 Constanta, Romania; 4 Department of Toxicology, Faculty of Pharmacy, Ovidius University of Constanta, 900470 Constanta, Romania; horatiu.miresan@univ-ovidius.ro (H.M.); irina.iancu@365.univ-ovidius.ro (I.M.I.)

**Keywords:** gut microbiome, dysbiosis, pharmacomicrobiomics, plant-derived foods and compounds, drug–microbiome interactions, molecular pathways, diagnostic methods

## Abstract

The digestive system is one of the most complex systems in the body, integrating multiple functions, closely linked to and influenced by chemosensory mechanisms, as well as by the presence, composition, and dynamics of the microbiome. Increasing attention has been directed toward plant-derived foods and medicines, which interact with gut microbiota and modulate host physiological responses through microbial metabolism, leading to the formation of bioactive metabolites that influence host signaling pathways and therapeutic response. The review, based on relevant articles from major international databases using specific terms with a focus on microbiome-mediated interactions and molecular mechanisms, highlights the role of microbiome and diagnostic methods through the analysis of specific composition and changes in microbiota, as well as the importance of microbiomes in relation to the treatment of chronic diseases, given their complex influence on drug metabolism. The microbiome influences the response to medications and resistance to therapy, being also involved in the metabolism of plant-derived foods and medicines through complex microbial interactions, while the importance of modern diagnostic approaches supports the use of microbiome analysis to improve diagnosis, monitoring, and personalized medical strategies.

## 1. Introduction

The gastrointestinal tract is a remarkable component of the body that performs multiple essential tasks, functioning in an integrated manner across various systems and over different time frames [1,2,3]. Recent advances have improved our understanding of how its specific cells, tissues, and organs collaborate to regulate digestion, absorption, and excretion of food, as well as to support immune, endocrine, and barrier functions [4,5]. All these processes depend on a highly integrated system, which includes the microbiota, a diverse community of bacteria, protozoa, viruses, archaea, and fungi, as well as myogenic, neural, humoral, and immune components that coordinate anatomical, endocrine, metabolic, and immunological processes [6,7].

The human gut is colonized by a considerable number of microorganisms that are deeply integrated into the host’s physiological functions [8,9,10]. Human microbiota consists of trillions of microorganisms that primarily colonize the gut, establish themselves after birth, and shape the development and maturation of the immune system [11,12]. Through its diversity, with over 100 bacterial phyla and approximately 150 times more genes than the human genome, it performs essential protective functions in a symbiotic relationship with the host [8,13]. In healthy individuals, the gut microbiota is dominated by the phyla Firmicutes and Bacteroidetes, which together represent over 90% of the microbial community, followed by Proteobacteria and Actinobacteria [13,14]. In addition to alpha diversity, which reflects microbial diversity within an individual, there is also significant beta diversity, representing variations in microbiota composition among different individuals [15,16].

The gastrointestinal lumen contains compounds derived from the diet and the microbiota, with potentially beneficial or harmful effects, which, upon ingestion, are unconsciously detected through specialized chemosensory mechanisms of the gastrointestinal tract, triggering appropriate physiological responses. This emphasizes the fact that the gastrointestinal tract serves not only as a site for digestion and absorption, but also as a complex chemosensory organ [17,18].

Recent studies have highlighted the role of microbial metabolites, such as short-chain fatty acids (SCFAs), bile acid derivatives, and tryptophan metabolites, in modulating host signaling pathways involved in inflammation, immunity, and the regulation of host energy metabolism, as well as in maintaining mucosal integrity. These metabolites are key mediators linking diet, the microbiota, and host physiology [19].

Recent developments in multi-omics technologies have significantly expanded the understanding of interactions between the host and the microbiome. This has allowed for the identification of functional microbial signatures associated with pathological conditions and therapeutic responses. These approaches provide a transition from descriptive analysis of microbiota to a mechanistic understanding of how microorganisms contribute to drug metabolism and efficacy [20].

At the same time, pharmacomicrobiomics has emerged as a distinct discipline that studies the bidirectional interactions between drugs and the microbiome. This discipline demonstrates that microbial enzymes can influence drug bioavailability, therapeutic outcomes, and toxicity, thereby contributing to interindividual variability in treatment response [21,22]. The gut microbiota can affect drug metabolism and toxicity indirectly through, for example, modulation of host drug metabolism and disposition and competition of bacterial-derived metabolites for xenobiotic metabolism pathways. In addition, therapeutic drugs themselves can exert both intended and unintended effects that impact the health and composition of the gut microbiota with potentially unforeseen consequences [23].

As therapeutic innovation continues to advance, there is a growing interest in microbiome-targeting interventions, including probiotics, prebiotics, postbiotics, fecal microbiota transplantation (FMT) [24,25], and strategies based on phytochemicals, which aim to restore microbial balance and improve therapeutic efficacy [26]. Phytochemicals are of relevance due to their anti-inflammatory, antioxidant, immunomodulatory [27,28] and metabolic effects; however, the physiological chemosensory mechanisms underlying their action remain only partially elucidated [29].

Dysbiosis of normal gut microbiota is associated, among other things, with digestive, metabolic, cardiovascular, immune, and neurological diseases [30,31]. Overall, microbiome-based diagnostics is an emerging field that utilizes microbial communities and their functional profiles to support disease detection, monitoring, and personalized medical care [32]. Furthermore, progress made in next-generation sequencing and machine learning (ML) algorithms has facilitated the development of microbiome-based diagnostic tools capable of predicting disease susceptibility and treatment outcomes with increasing accuracy. These diagnostic platforms integrate microbial composition with functional and metabolic data, supporting precision medicine approaches [33,34].

The gut microbiota has become a key element in regulating host physiology, but the integrative mechanisms linking chemosensory pathways, microbial metabolism, and host signaling are not yet fully understood. At the same time, growing concerns regarding antibiotic resistance and variability in therapeutic response highlight the need for microbiome-based strategies.

This article focuses on information regarding the molecular mechanisms triggered by interactions between the microbiome and chemical compounds or micronutrients that enter the digestive tract. It accentuates recent discoveries regarding food, phytochemical composition, and signaling pathways that influence health. It also notes the importance of understanding microbiota in a therapeutic context and in personalized approaches to diagnosis and treatment planning.

The importance of biologically active substances, particularly phytochemicals, has become a subject of interest with the development of knowledge in nutrition and dietetics, an independent scientific field that has focused on the physiologically active properties of nutrients. Pharmacology and pharmacognosy contribute to the advancement of this field, considering the substantial increase in using pharmacologically active natural products in recent years. Consequently, the safe use of these natural products requires a comprehensive understanding of phytochemistry and molecular biology, as interactions with the microbiome are essential.

Therefore, a comprehensive approach that integrates microbial, molecular, and therapeutic data is essential for progress in this field.

## 2. Intestinal Microbiota, Dysbiosis and Pathological Implications

### 2.1. Microbiota—Normal Status and Changed Prospective

Intestinal microbial colonization begins at birth and evolves through a succession of taxonomic changes until a balanced diversity is reached, like that of adults [35,36]. After childhood, the proportion of different species remains relatively stable throughout life, although in the elderly, a shift in dominance from Firmicutes toward Proteobacteria and *Alistipes* has been observed [37,38].

The gastrointestinal tract possesses chemosensory pathways similar to those involved in taste and smell, which, through an integrated network of epithelial cells, receptors, and neural circuits, continuously monitor luminal contents to maintain energy homeostasis, interpret food signals, coordinate metabolic responses, and communicate with the brain via the enteric nervous system, being essential for assessing the biological value of ingested food [39,40]. Chemosensing in the gastrointestinal tract extends beyond a strictly digestive role, influencing appetite, energy metabolism, immunity, and brain activity via the gut–brain axis of the microbiota [41,42,43].

In this context, recent research has highlighted the varied roles of gut microbiota in energy metabolism, in regulating immune responses, and in protection against pathogens [44,45]. The microbiota acts as a competitive barrier against pathogenic microbes [46,47], contributing to the preservation of the intestinal mucosal barrier and the overall health [48,49], while also regulating the balance between pro-inflammatory and anti-inflammatory responses [50,51]. The neutralization of pathogens is also supported by the release of antimicrobial metabolites [52,53].

**Figure 1:**
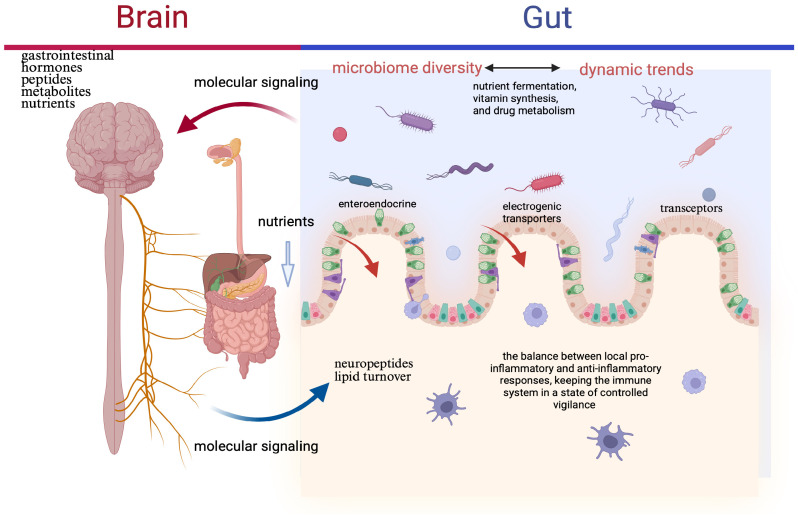
Interactions between brain and gut signaling and microbiome diversity (created in https://BioRender.com).

Moreover, microbiota is involved in nutrient fermentation, vitamin synthesis, and drug metabolism, as well as in the supply of essential nutrients, including short-chain fatty acids, to colonic epithelial cells [54,55]. Products of microbial metabolism, identified in biological compartments such as feces, urine, and cerebrospinal fluid, reflect the close interaction between the host and the microbiome [56,57,58], while microbiota also play roles in brain development and function, highlighting the relevance of the gut–brain axis (Figure 1) [59,60].

**Figure 2:**
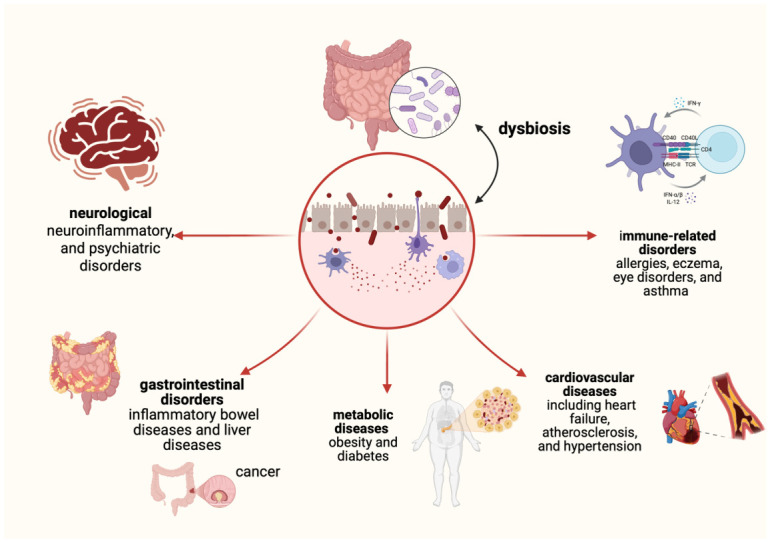
Dysbiosis and associated pathologies (created in https://BioRender.com).

In this context, dysbiosis is defined as an alteration in the composition and function of the microbiota [61,62] and is recognized as a feature associated with health, aging and disease, contributing to the concept of the host as a “meta-host” [63,64]. Experimental studies in animal models have demonstrated not only associations, but also a potential causal relationship between microbiome alterations and systemic diseases (Figure 2) [65,66].

Dysbiosis has been associated with neurological, neuroinflammatory, and psychiatric disorders, including autism disorder, anxiety, depression, Parkinson’s disease, Alzheimer’s disease, multiple sclerosis, stress, and addiction [67,68], as well as with gastrointestinal disorders [69,70], particularly inflammatory bowel diseases and liver diseases [71,72]. In addition, microbiota alterations are linked to metabolic diseases (Figure 2), such as obesity and diabetes; cardiovascular diseases, including heart failure, atherosclerosis, and hypertension [73,74]; immune-related disorders such as allergies, eczema and asthma (Figure 2) [75,76]; and oncological diseases, including colorectal cancer, hepatocellular carcinoma, cholangiocarcinoma, pancreatic ductal adenocarcinoma and gastric cancer [77,78].

Furthermore, several host factors influence the physiological state of the gut microbiota and contribute to disease development. In early life, gut microbiota play an essential role in immune system maturation, and early dysbiosis has been associated with allergies, asthma, and obesity [79], while dietary patterns such as a high-fat diet (HFD) are associated with metabolic diseases and specific alterations in microbiome composition and function [80,81].

In oncology, the mechanisms by which microbiota contribute to carcinogenesis are increasingly characterized. The *Fusobacterium nucleatum* adhesin A (FadA) antigen promotes colorectal cancer (CRC) by activating the E-cadherin-Wnt-β-catenin signaling pathway; the genotoxin colibactin produced by PKS-positive strains of *Escherichia coli* amplifies colorectal tumorigenesis; and the toxin from *Bacteroides fragilis* generates reactive oxygen species (ROS), causing DNA damage in malignant gastrointestinal tumors [82,83,84]. Although these bacteria have direct effects on tumorigenesis, some microbes promote inflammation or weaken immune surveillance, indirectly facilitating cancer development—mechanisms conceptually integrated into the “immuno–oncology–microbiome axis” [85,86].

Several studies demonstrated that gut microbiomes can directly influence an individual’s response to a specific drug by enzymatically altering its structure (Table 1). As a result, the drug’s bioavailability, bioactivity, or toxicity may be modified, a phenomenon termed pharmacomicrobiomics [21,87].

Next-generation sequencing, particularly 16S rRNA gene analysis, has shown that antibiotics disrupt the composition of gut microbial communities, which has direct effects on microbiome–drug and microbiome–phytochemical interactions [88,89]. Combined microbiome–metabolomic analysis has identified correlations between microbial changes and the host’s metabolomic profile [90,91]. Several classes of drugs (proton pump inhibitors, metformin, and statins) show consistent associations with specific microbial signatures, reproducible in independent cohorts [92,93,94,95,96,97]. Antibiotic-induced dysbiosis, manifested by a decrease in microbial diversity and changes in dominant phyla, can disrupt the biotransformation of plant-derived compounds and thus reduce the therapeutic efficacy of phytomedicines [98,99,100].

### 2.2. Host–Microbiome Interactions and Major Mechanisms of Modulation

In gastrointestinal pathology, association studies have demonstrated specific dysbiotic profiles [101,102], highlighting the role of microbiota in colonization resistance and prevention of enteric infections [103,104]. In inflammatory bowel diseases, increases in adherent-invasive *Escherichia coli* strains [105,106] and reductions in butyrate-producing bacteria have been associated with inflammatory processes [107,108]. Similarly, in irritable bowel syndrome and metabolic diseases, alterations in microbiota diversity and composition have been reported, although the heterogeneity of results limits their use as robust diagnostic biomarkers [109,110,111,112,113].

In parallel, specific microbial signatures have been associated with cardiovascular [114,115] and allergic diseases, including correlations between Enterobacteriaceae and cardiovascular risk, as well as associations between early-life microbiota composition and allergic sensitization [116,117]. In addition, reductions in immunomodulatory [118,119] and butyrate-producing bacteria have been linked to increased allergic risk and impaired immune tolerance [120,121]. Furthermore, in neurodegenerative pathology, patients with Alzheimer’s disease have been shown to present reduced microbiota diversity and specific compositional changes [122,123], supporting the involvement of the gut–brain axis, although clinical applicability requires further validation [124].

Overall, microbiome-based diagnostics is an emerging field that utilizes microbial communities and their functional profiles to support disease detection, monitoring, and personalized medical care [32]. From a therapeutic perspective, the microbiota’s influence on host physiology is primarily mediated by microbial metabolites that activate specific receptors [125,126].

Several factors associated with the host organism (physiology, metabolic stress, host immunity) are involved in mechanisms that modulate the microbiome. Furthermore, recent studies highlight the interactions between microbiome and gene regulation in host cells, suggesting major and potentially irreversible effects. The physiology of microorganisms, the response mediated by the release of bacterial enzymes, and the genetic adaptability of the microbiome are significantly involved in host physiological processes such as steroid biosynthesis, bile production, and interactions with the metabolism of substances derived from the ingestion of drugs or xenobiotics (Table 1).

**Table 1:** The host mechanisms and microbial activity after interactions with inductor factors.

Crt. No.	Inductor Factors	Major Mechanism and Microbial Modulation	References
1.	Stress-associated signaling molecules	Synthesize and take up catecholamine and interference with glucocorticoid activity *Clostridium scindens*, a reference model for studying bacterial steroid metabolism *Ruminococcus gnavus*, correlating positively with cortisol concentrations, was identified as a cortisol metabolizer	[127]
2.	Bacterial CYP450 enzymes	steroid biosynthesis, bile acid production, drug-xenobiotic metabolism	[128]
3.	Genetics of microbiome and host interactions	Gut microbiome-associated genetic variant (MAV) disease and host gene regulation; epigenetic and transcriptional changes	[129,130,131]
4.	Host immune system	Regulatory T cells and macrophage epigenetic regulation of cytokine expression and HDAC inhibition, acetylation of FoxP3 CNS1 region, induction of proliferation, upregulation of Uhrf1 T helper 17 cell interaction with transcriptional reprogramming through epithelially produced SAA1 and SAA2; Natural killer T-cell and mucosal recruitment via CXCL16	[131]
5.	Specific drugs		
	Anti-inflammatory treatment	Gram-negative bacteria increased and epithelial disequilibrium	[132]
	Cardiac glycosides	Enzymatic interactions and drug metabolism suppressed	[132]
	Metformin	Drug effects inhibitions	[132]
	Chemotherapeutics	Multiple changed and negative drug effects	[132]
	Antibiotics	Microbiome diversity affected	[132,133,134]
6.	Phytochemicals (alkaloids, flavonoids, saponins, polysaccharides and betacyanins)	Formation of bioactive metabolites through interaction with the microbiota → regulation of AMPK, NF-κB, mTOR, PPAR, cytokine, and SCFA pathways Microbial transformation of polyphenols → alteration of microbiota composition Flavonoids → phenolic metabolites + bidirectional effect on the microbiota (structure/diversity) Polyphenols → antioxidant, anti-inflammatory, prebiotic effects + stimulation of beneficial bacteria (*Bifidobacterium*, *Akkermansia*) Alkaloids → nonspecific antimicrobial effect → possible decrease in diversity	[26,84,135,136,137,138,139,140,141,142,143,144,145,146,147,148,149]
7.	Dietary supplements (with berberine, curcumin, spirulina, plant extracts rich in polyphenols)	Variable microbiota–supplement interactions (beneficial effects/dysbiosis) Berberine → ↑ SCFA-producing bacteria + ↑ GLP-1 Curcumin → ↑ *Lactobacillus*, *Bifidobacterium* Spirulina → antimicrobial effect + support for probiotic bacteria Polyphenolic supplements → substrate for beneficial microbiota (prebiotic effect)	[140,150,151,152,153,154,155,156,157]

HDAC—histone deacetylase; CYP450—enzymes catalyze the biotransformation and clearance of a wide array of xenobiotics and potentially toxic compounds; FoxP3 CNS1 (Conserved Non-coding Sequence 1)—protein (scurfin) involved in immune system responses, transcriptional regulator; SAA1, SAA2—the human protein family (circulating serum amyloid A) comprises the acute phase SAA1/SAA2, known to activate a large set of innate and adaptive immune cells; CXCL16—chemokine ligand 16; AMPK—AMP-activated Protein Kinase; NF-κB—nuclear factor kappa B pathway is a crucial signaling mechanism that regulates innate immunity, inflammation, cell survival, and differentiation; mTOR—the mammalian target of rapamycin (mTOR) pathway is a central signaling hub that regulates cell growth, proliferation, metabolism, and survival in response to nutrients, energy levels, and growth factors; PPAR—Peroxisome Proliferator-Activated Receptors (PPARs) are nuclear receptor proteins that act as ligand-activated transcription factors to regulate gene expression involved in lipid/carbohydrate metabolism, cellular differentiation, and inflammation; SCFA—Short-chain fatty acids, primarily acetate, propionate, and butyrate are produced via anaerobic bacterial fermentation of dietary fibers, resistant starches, and amino acids in the gut; GLP-1—glucagon-like peptide-1.

Various chemical compounds derived from specific therapies for chronic diseases, as well as phytochemicals from diverse sources, whether through diet or alternative medicine, aromatherapy, or herbal medicine, have been closely studied in recent years to understand the links between composition, mechanisms, and induced chemical reactions with varied effects on the microbiota. Elucidating all cause-and-effect relationships within this research framework remains an ongoing goal.

In this context, based on the analysis conducted in this review, particular attention is drawn to the main classes of microbial metabolites implicated as therapeutic targets: short-chain fatty acids (SCFAs), bile acids, and tryptophan derivatives. These metabolites interact with G protein-coupled receptors, as well as taste and olfactory receptors, to modulate health status [158,159]. Acetate, propionate, and butyrate, produced by the fermentation of dietary fiber, act as ligands for G protein-coupled receptors (FFA2, FFA3, GPR109A) and for olfactory receptors (Olfr78, OR51E2) [160,161], leading to the activation of signaling pathways that regulate the secretion of intestinal peptide hormones, blood pressure, renin secretion, airway contraction, and the expression of antimicrobial peptides [162,163]. Overall, short-chain fatty acids increase satiety, thermogenesis, fatty acid oxidation, glucose metabolism regulation, and the secretion of intestinal hormones, thereby supporting their therapeutic potential in obesity and metabolic diseases [164,165].

## 3. Microbiome and Food Interactions

### 3.1. General Pathways and Molecular Signaling

Intestinal epithelial cells express taste, olfactory, and microbial metabolite-sensitive receptors, which enable the detection of food and microbial compounds and support the initiation of appropriate protective responses [125,166,167].

The gut microbiome is highly dependent on diet, particularly dietary fiber intake, which significantly influences the composition of this microbial community; it includes certain gut microbial groups equipped with a broad enzymatic repertoire capable of degrading complex dietary polysaccharides, such as pectins, xylans, fructans, starch, and arabinogalactans [168,169].

Species belonging to the genera *Bifidobacterium* and *Bacteroides*, recognized as the primary degraders of these polysaccharides, exhibit molecular mechanisms that have been partially elucidated [170,171], while the utilization of plant-derived oligosaccharides is common among gut microbes, reflecting molecular adaptations to exploit complex substrates [172,173]. By fermenting these substrates, the microbiota produces bioactive metabolites, establishing a link between diet, microbiome, and therapeutic response, particularly in metabolic syndrome and inflammatory bowel diseases.

In parallel, bile acids act as signaling molecules via the farnesoid X nuclear receptor (FXR) and the Takeda G-protein-coupled receptor 5 (TGR5) [174,175], while microbial conversion of primary into secondary bile acids modifies receptor specificity and shapes metabolic and immune responses. Deoxycholic acid (DCA), a potent TGR5 agonist, stimulates GLP-1 secretion, improves insulin sensitivity, increases energy expenditure, and reduces inflammation [176,177].

**Figure 3:**
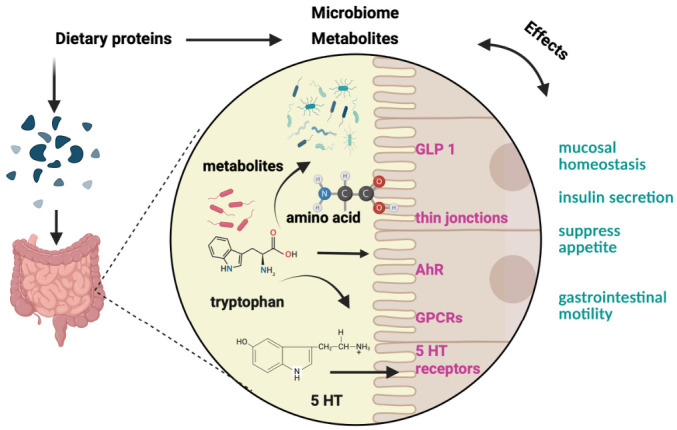
The protein dietary, molecular pathways and typical effects; GLP 1—Glucagon-Like Peptide-1; AhR—Aryl Hydrocarbon Receptor; GPCRs—G Protein-Coupled Receptors; 5 HT—5-Hydroxytryptamine (Serotonin). Created in https://BioRender.com.

Proteins from various sources (animals, plants, marine organisms) form diverse combinations and, together with other macronutrients, can have synergistic or antagonistic effects on the gut microbiota. Proteins undergo progressive biochemical changes throughout the digestive tract; following the action of proteolytic enzymes, any residual undigested proteins are subjected to bacterial hydrolysis, resulting in the release of amino acids. Amino acids can serve as building blocks for bacterial components or be converted into metabolites (SCFAs, polyamines, hydrogen sulfide, phenols, and indoles), and the resulting metabolites may be involved in various physiological functions related to the host’s health (Figure 3) [178].

Furthermore, dietary tryptophan (Trp) is metabolized by the gut microbiota into indoles (indolepropionic acid, indoleacetic acid, indolealdehyde), tryptamine, and other aromatic compounds [178,179], which act on the aryl hydrocarbon receptor (AhR), the pregnane X receptor, GPCRs, GLP 1 and 5 HT (serotonin) receptors (Figure 3). Indole derivatives activate AhR to stimulate IL-22 production and tight junction expression [180,181].

Furthermore, microbiota-mediated regulation of serotonin synthesis links tryptophan metabolism to intestinal motility and systemic signaling [182,183]. Short-chain fatty acids (SCFAs), bile acids, and tryptophan derivatives exemplify how microbiota-derived metabolites act via chemoreceptors expressed by epithelial, immune, and neural cells [184,185], integrating metabolic, immune, and neuroendocrine functions and supporting therapeutic opportunities in disorders associated with dysbiosis [186,187].

Eating habits and dietary patterns vary greatly, with a preference for diets rich in animal protein in many modernized areas, often associated with the consumption of unhealthy, ultra-processed foods. Excess protein can promote the growth of pathogenic microorganisms and increase the production of toxic microbial metabolites (hydrogen sulfide, ammonia, indole compounds), which have harmful effects by increasing the risk of metabolic and kidney diseases [188]. A diet rich in animal protein is also associated with a selective decrease in fiber-fermenting *Firmicutes* such as *Roseburia*, *Eubacterium rectale*, and *Ruminococcus bromii*, alongside the expansion of bile-tolerant bacteria such as *Alistipes*, *Bacteroides*, and *Bilophila*, a microbial shift linked with inflammatory bowel conditions [189].

**Figure 4:**
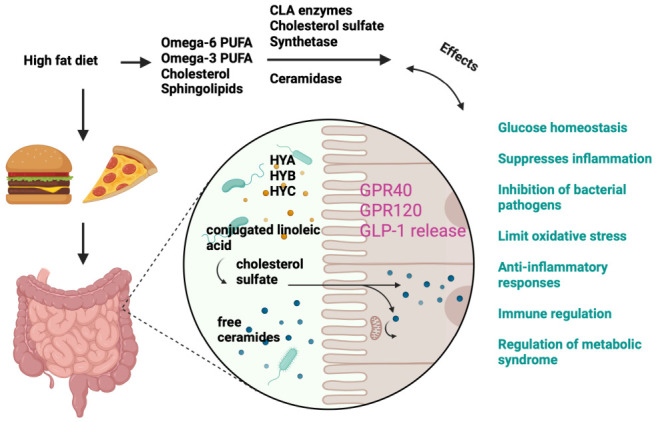
The high-fat diet, molecular activation pathways and their effects. PUFA—Polyunsaturated Fatty Acid; CLA—Conjugated Linoleic Acid; HYA—10-Hydroxy-cis-12-octadecenoic acid; HYB—10-Hydroxy-12(Z)-octadecenoic acid; HYC—10-Hydroxy-12(E)-octadecenoic acid; GPR40—G Protein-Coupled Receptor 40 (FFAR1); GPR120—G Protein-Coupled Receptor 120 (FFAR14); GLP-1—Glucagon-Like Peptide-1. Created in https://BioRender.com.

The main classes of lipids ingested by humans are metabolized and biotransformed by the gut microbiome. The subsequent impact on the host’s immune and metabolic pathways is driven by the presence of the metabolites generated, as well as by the activation of host cell signaling (Figure 4) through the activity of G protein-coupled receptors (GPR40, GPR120, GLP 1) and complex effect response pathways [190].

A high-fat diet has harmful effects on intestinal permeability and mucus layer integrity, while impairing metabolic functions. It is associated with changes in microbiota composition and increased production of endotoxins, which promote inflammation and metabolic disorders. Studies have reported an increase in *Firmicutes* and a decrease in *Bacteroidetes* in association with high-fat diets. Furthermore, high intake of fatty acids is linked to ulcerative colitis and an increased risk of colon cancer [191]. Campaniello D. et al. showed that high fat intake stimulates the gut microbiota to produce endotoxins (lipopolysaccharides), creating a favorable environment for the proliferation of Gram-negative bacteria, particularly Enterobacteriaceae, resulting in increased inflammation and intestinal permeability and contributing to obesity development [192].

**Figure 5:**
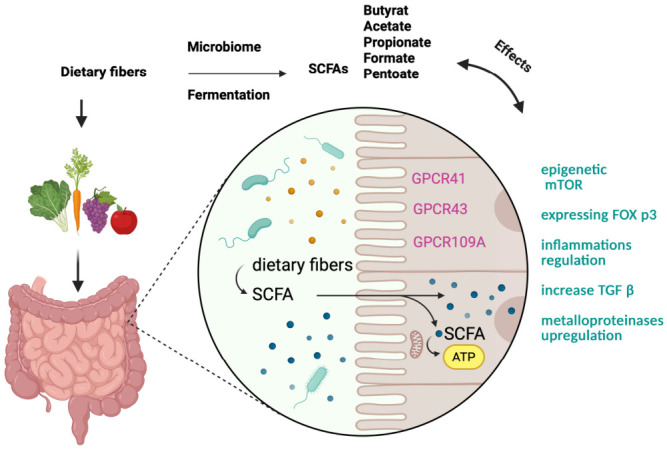
Dietary carbohydrates and the implications of SCFA metabolite production. SCFAs—Short-Chain Fatty Acids; GPCR41—G Protein-Coupled Receptor 41 (FFAR3); GPCR43—G Protein-Coupled Receptor 43 (FFAR2); GPCR109A—G Protein-Coupled Receptor 109A (HCAR2); ATP—Adenosine Triphosphate; mTOR—the mammalian target of rapamycin; Fox p3—protein (scurfin) involved in immune system responses, transcriptional regulator; TGF β—transforming growth factor is a multifunctional cytokine expressed by almost every tissue and cell type. Created in https://BioRender.com.

Complex carbohydrates, particularly dietary fiber, undergo microbial fermentation in the colon, resulting in the production of short-chain fatty acids (SCFAs) (Figure 5) [193].

SCFAs influence appetite regulation, modulate regulatory T-cell function and immune responses and affect lipid and glucose metabolism [194]. There is also the upregulation of the expression of certain factors (mTOR, FOX p3, TGF) for the regulation of cellular processes (Figure 5) in both the colon and other tissues [195].

These compounds impact the gastrointestinal microbiome by preventing pathogen adhesion to the intestinal mucosa [188]. A diet rich in dietary fiber reduces insulin resistance in patients with type 2 diabetes [191] and decreases the incidence of gastrointestinal diseases, including irritable bowel syndrome, inflammatory bowel disease, colorectal and gastric cancer, and constipation [194]. The Mediterranean diet promotes the increase in health-associated gut bacteria, including genera such as *Bifidobacterium*, *Lactobacillus*, *Faecalibacterium* (e.g., *F. prausnitzii*), *Roseburia*, and members of the *Lachnospiraceae* family, alongside a reduction in potentially pathogenic taxa belonging to Proteobacteria, Enterobacteriaceae, and certain *Clostridium* and *Blautia* species [194,196,197,198,199,200,201].

Similarly, the vegetarian/vegan diet is characterized by the consumption of plant-based foods, with a high intake of fruits, vegetables, grains, wine, tea, and coffee, which are rich in carbohydrates, fiber, and phytochemicals. The microbiota associated with this diet is abundant in *Prevotella* and reduced in *Bacteroides* [199,202,203].

Moreover, high intake of fiber and polyphenols (flavonoids, phenolic acids, stilbenes, lignans) led to an increase in *Bifidobacterium* spp., *Lactobacillus* spp., *Ruminococcus* spp., *Eubacterium rectale*, and *Roseburia* spp., which provided cardiovascular, antibacterial, and anti-inflammatory protection [194,202].

The ketogenic diet influences the gut microbiota through an increase in *Firmicutes*, *Enterobacteria*, *Desulfovibrio* spp., *Parabacteroides*, *Bacteroidetes*, *Actinobacteria*, and *Akkermansia*, and a decrease in *Bifidobacteria*, *Eubacterium rectale*, *Dialister*, and Proteobacteria [194,199,200,204]. Research by Rew L. et al. (2022) [205] and Perler B. et al. (2023) [196] showed that adherence to a ketogenic diet alters microbial composition and metabolites, notably decreasing *Bifidobacterium* spp. and reducing fecal short-chain fatty acids, including butyric acid, propionic acid, and acetic acids, with negative effects on colon health.

The gluten-free diet is a therapeutic option for the management of celiac disease. The study by Campaniello et al. [192] highlighted that a gluten-free diet may also reduce beneficial bacteria such as *Lactobacillus*, *Bifidobacterium*, and *Roseburia*, while increasing *Escherichia coli* and Enterobacteriaceae, Victivallaceae, and Clostridiaceae, indicating potential negative effects on microbiota balance in healthy individuals. Work by Delmas et al. [206] demonstrated that a prolonged gluten restriction has been associated with reduced microbial diversity, decreased abundance of beneficial species such as *Akkermansia muciniphila* and *Bifidobacterium* spp., disruption of fiber-degrading and butyrate-producing communities, and functional shifts in microbial metabolism.

### 3.2. Plant-Derived Compounds and Microbiome Interactions

The consumption of foods, dietary supplements, or herbal medicines provides a supply of plant-based substances that interact with gut microorganisms. In this context, microbial consortia transform complex plant-based compounds into structurally diverse metabolites that differ in availability and bioactivity, while also influencing the gut ecosystem by modulating microbial composition [138,140].

Biologically active substances of plant origin belong to various classes of compounds based on their chemical composition and structure, including linear, aromatic, and heterocyclic structures; often containing one or more functional groups, frequently mixed, that facilitate diverse interactions.

A classification of phytochemicals is presented to support their further discussion; in this regard, seven classes of biologically active substances are mentioned, such as organic acids, alkaloids, phytoncides, heterosides, natural pigments, tannins, and essential oils.

The importance of these compounds for microbiota is also related to the fact that, in the intestinal tract, 95% of phytochemicals pass through the small intestine unabsorbed, meaning that their major influence is found in the colon. At this level, microbiota (*Escherichia coli*, *Bifidobacterium* sp., *Lactobacillus* sp., *Bacteroides* sp., and *Eubacterium* sp.) metabolize plant compounds into bioactive metabolites [207].

Maternal nutrition during gestation and lactation shapes both immediate and long-term offspring health by directing growth, metabolic phenotypes and gut microbiome development; in this context, maternal malic acid consumption remodeled the microbial community of piglets, increasing the abundance of *Megasphaera*, *Romboutsia*, *Colidextribacter*, and *Cloacibacillus*, while reducing *Prevotella*, *Blautia*, *Faecalibacterium*, Prevotellaceae, *Collinsella*, Butyricicoccaceae, Lachnospiraceae, and *Olsenella*, a change that may influence skeletal muscle function and metabolic outcomes [208].

Berberine exerts anti-inflammatory effects in part by activating the TLR4/MyD88/NF-кB signaling axis and modulates gut microbiota, increasing *Bacteroidetes*, *Clostridia*, *Lactobacillales*, Prevotellaceae, *Alloprevotella*, *Butyricimonas*, *Coprococcus*, *Ruminococcus*, *Lactobacillus*, *Akkermansia*, *Verrucomicrobia*, while reducing *Bacteroidales*, Lachnospiraceae, Rikenellaceae, *Desulfovibrio*, Streptococcaceae, Clostridiaceae, *Prevotella*, *Proteus*, *Saccharibacteria*, *Deferribacteres*, *Actinobacteria*, changes that are implicated in diabetes pathogenesis. Additionally, gut microbiota metabolizes berberine in dihydroberberine, which reduces intestinal disaccharide absorption, stimulates GLP-1 and GLP-2 secretion to preserve pancreatic islet function and lowers blood glucose levels [153].

Dietary factors may contribute to interindividual variability in digoxin reduction: arginine stimulates human gut bacteria *Eggerthella lenta* growth, while suppressing cgr operon expression, preventing digoxin in vitro conversion to dihydrodigoxin and in vivo studies on gnotobiotic mice reveal that high-protein diets diminish digoxin microbial metabolism, leading to changes in serum and urinary drug concentrations [209].

Carotenoids, especially astaxanthin and retinoic acid, help maintain gut immune balance by enhancing IgA production, limiting epithelial penetration and preventing dysbiosis. Also, supplementation of lycopene administered to obese adults revealed an increased abundance of *Bifidobacterium adolescentis* and *Bifidobacterium longum*, and *Lactobacilli* in a dose-dependent manner, alongside a reduction in low-density lipoprotein cholesterol (LDL-C), LDL-peroxidase, and malondialdehyde (MDA), thus indicating an improvement in oxidative stress levels [210].

The microbial biotransformation of condensed tannins by bacterial species such as *Eubacterium cellulosolvens*, *Bacteroides distasonis*, *B. ovatus*, *B. uniformis*, *Enterococcus casseliflavus*, *Eubacterium ramulus*, and members of the Lachnospiraceae family (CG191) results in the production of pharmacologically active metabolites. These compounds, through mechanisms such as the activation of CD4^+^ cells and natural killer cells, the inhibition of metalloproteinases MMP-1, MMP-2, and MMP-9, the suppression of nitric oxide production by blocking inducible NO synthase (iNOS), modulation of the inflammatory response by inhibiting TNF-α and the NF-κB–IKK–IκBα cascade, as well as by reducing the expression of the endothelial adhesion molecules such as vascular cell adhesion molecule 1 (VCAM-1) and monocyte chemotactic protein-1 (MCP-1), converge toward phenolic structure-dependent antioxidant, anti-inflammatory, and anti-atherosclerotic effects [211].

Taken together, these examples and other phytochemicals synthesized in Table 2 highlight the diversity of molecular pathways through which plant-derived compounds shape the gut microbiota.

Verediano et al. [218] conducted a systematic review that highlights how anthocyanin supplementation effectively modulates gut microbiota by increasing *Lactobacillus* and *Bifidobacterium* populations and promotes short-chain fatty acid (SCFA) production. SCFAs can activate colon NLRP6 (nucleotide-oligomerization domain-like receptor 6), thereby enhancing intestinal barrier protection. SCFAs also promote colonic epithelial renewal and mucus secretion, activate the mammalian target of rapamycin (mTOR) complex and the signal transducers and activator of transcription 3 (STAT3), which upregulates antimicrobial peptides (e.g., β-defensin, RegIIIγ). Additionally, SCFAs exert immunomodulatory effects by stimulating mucosal regulatory T-cell (Treg) development through interaction with G protein-coupled receptor (GRP43) and histone deacetylases (HDACs) inhibition.

There are generally few systematic studies on the interaction between phytochemicals and colon cells, and there is also little data on the molecular mechanisms involved.

Phytoconstituents (alkaloids, flavonoids, saponins, polysaccharides and betacyanins) undergo dynamic interactions within the gut microbiota (Table 2), forming diverse and bioactive metabolites [26] that significantly influence cellular signaling pathways (e.g., AMPK, PPARs, MAPK, cellular redox balance, sirtuins, TLR4, NF-κB, mTOR, SCFAs, IL-6, JNK and TNF), host metabolism and immune responses [27,135].

Flavonoids can have significant effects on microbiota composition under experimental conditions, influencing microbial structures and diversity as part of a bidirectional interaction, with modulatory effects that may be positive or negative rather than universally reducing diversity [141]. The colonic microbiota converts flavonoids into metabolites such as phenolic acids, and this selective processing may influence microbial community dynamics and host physiology [139]. Several studies suggest that compounds such as quercetin may inhibit the growth of certain bacteria under specific conditions and at higher concentrations, although this effect depends on chemical structure and dose [222]. In addition, flavonoids can alter microbial structure, including increasing certain bacteria (e.g., Proteobacteria) associated with dysbiosis, which may reduce functional diversity or disrupt the bacterial community under specific conditions [141,157].

Similarly, polyphenols exert antimicrobial activity and influence the composition and functionality of the gut microbiota [142], while also acting as antioxidants [223,224], with anti-inflammatory and prebiotic effects [143,225]. Their metabolism by gut bacteria leads to the production of microbial metabolites, such as organic acids, which can alter pH and influence microbial enzymes and microbiota composition [149]. Fermentation mediated by polyphenolic metabolites exerts selective pressures on the gut microbiota, as commensals and pathogens differ in their ability to utilize these metabolites or respond to their antimicrobial effects [144,145]. In this context, polyphenols and their metabolites can stimulate the growth of beneficial bacteria, such as *Bifidobacterium* and *Akkermansia muciniphila*, while inhibiting pathogenic species [146].

In contrast, alkaloids, although natural plant compounds, may have adverse effects on gut microbiota when consumed frequently or at high doses, due to their non-selective antimicrobial properties, which may also affect beneficial bacteria and reduce microbiota diversity over time [147,148].

The relationship between the microbiome and the biological effects of metabolites of tannins biotransformed in the intestine, as well as molecular signaling, remains largely unexplored. The link between their chemical structures and their potential mechanism of action is important, as it facilitates the discovery of specific molecular characteristics underlying the heterogeneous information associated with pharmacological effects [211].

Certain herbal supplements interact significantly with microbiota (Table 2), but their impact can be variable and is not fully understood, with potential microbial imbalances, as evidence suggests both beneficial effects and the possibility of dysbiosis [150,151].

In this context, berberine supplements modulate the composition of the gut microbiota, increasing the proportion of short-chain fatty acid-producing bacteria (including *Bacteroides*, *Blautia*, *Butyricicoccus* and *Phascolarctobacterium*) and promoting the expression of glucagon-like peptide-1 (GLP-1) in intestinal L cells [152,153]. Similarly, turmeric (extracts of *Curcuma longa* and its curcuminoids) interacts with the microbiota, with clinical and experimental studies reporting changes in bacterial composition and increases in beneficial bacteria, such as *Lactobacillus* and *Bifidobacterium* [154]. In addition, supplements such as spirulina contain compounds with antimicrobial activity and can influence microbial populations by inhibiting undesirable bacteria and, under certain conditions, stimulating the growth of probiotic bacteria, while polysaccharides and other bioactive compounds contribute to microbiota modulation [155,156]. Furthermore, plants rich in polyphenols and flavonoids (e.g., green tea, rosemary, cinnamon) serve as substrates for beneficial gut bacteria and may stimulate microbial diversity or metabolic activity, acting as modulators of host–microbiome interactions [140,157].

### 3.3. Clinical Translational Challenges of Plant-Derived Medicines: From Animal Models to Human Therapy

Although preclinical evidence indicates that plant-based foods and medicines can favorably modulate the gut microbiota, the translation of these results into clinically validated therapeutic strategies remains, for now, limited [226]. In vivo studies report promising effects of phytochemicals and plant extracts on microbiota-associated disorders, but the ability of animal models to predict human responses remains limited, as differences between species in microbiome composition, host physiology, metabolism, and environmental exposures are too great to be ignored [227]. Microbiome-targeted therapies also face serious translational and regulatory hurdles: mechanisms of action remain difficult to define, quality control standards are insufficiently established, and clinical efficacy varies across populations [228].

A major limitation remains the compositional and functional differences between rodent and human microbiota. Murine models raised under germ-free or conventional conditions have clarified causal relationships between microbial taxa and host phenotypes, but the microbial ecology of laboratory mice differs markedly from that of humans in terms of the proportions of dominant phyla, metabolic capacity, and colonization history [229,230]. Bioactive metabolites produced from phytochemicals in the animal microbiota may differ considerably from those generated in the human colon, which limits the direct extrapolation of mechanistic data obtained in preclinical models to human physiology; in animal studies, two-week treatment with plant extracts was shown to change gut microbiota composition, caecum metabolome, and markers of lipid metabolism in ob/ob mice, a genetic model of leptin-deficient obesity and gut microbiota dysbiosis [231]. While in human interventional studies, the relationship between plant-based dietary interventions and gut microbiome composition has shown promising but variable results depending on baseline microbiota [232]. The conversion of polyphenols (ellagitannins to urolithins, isoflavones to equol) depends on the composition of the individual microbiome and varies greatly among humans. This variability is not captured in standardized animal experiments [233,234].

The variability of the human gut microbiome complicates the clinical translation of interactions between phytomedicine and the microbiome, as the response to plant compounds dependent on microbial biotransformation, such as the conversion of lignans by *Clostridium scindens* [235] or the deglycosylation of flavonoid glycosides by beta-glucosidase producing *Lactobacillus* and *Bifidobacterium* species, varies among individuals depending on microbiome composition, diet, age, and disease status [236].

Variability in the human gut microbiome can influence the biotransformation, bioavailability, and therapeutic efficacy of plant compounds [227], and this variability limits the generalizability of small-scale clinical studies, underscoring the need for stratification of participants according to microbiome enterotypes or functional profiles prior to phytochemical interventions [237].

Bioavailability and pharmacokinetics remain a serious translational barrier, as many phytochemicals active in cell cultures or animal studies have low oral bioavailability in humans due to rapid metabolism, low solubility, or first-pass clearance [238,239]. Curcumin, for example, has very low systemic bioavailability in humans, despite its well-documented anti-inflammatory and microbiome-modulating properties in preclinical models, except in specialized formulations: phospholipid complexes, nanoparticles, or co-administration with piperine [240,241], while therapeutic concentrations of berberine achieved in mice are difficult to replicate in humans due to rapid intestinal inactivation and hepatic extraction [242]. These limitations necessitate targeted delivery systems and dosing protocols adapted to human pharmacokinetics; preclinical evidence from animal models supports the role of plant-derived compounds in modulating gut microbiota and associated metabolic pathways, but these findings require validation in well-controlled human trials before causal claims can be made [243].

Methodological standardization remains a challenge in clinical studies of phytomedicine as well, as extraction methods, routes of administration, treatment duration, outcome measures, and microbiome assessment techniques (16S rRNA amplicon sequencing versus shotgun metagenomics) vary so widely across studies that data synthesis becomes difficult, and conflicting results are inevitable [244]. The lack of harmonized protocols for reporting microbiome changes following phytochemical interventions also hinders meta-analysis efforts; while initiatives such as the MIxS standard and the STORMS checklist are concrete steps toward standardization, their adoption in phytomedicine research remains limited [245].

Safety and regulation present their own obstacles. Phytomedicines are often perceived as inherently harmless [246], but many plant compounds can be hepatotoxic, nephrotoxic, or immunomodulatory at high doses or in vulnerable patients, particularly those with an intestinal barrier compromised by dysbiosis [193]. Phytochemicals can interact with concomitantly administered medications by modulating CYP450 or inhibiting P-glycoprotein, altering bioavailability and increasing the risk of adverse reactions, for example, *Hypericum perforatum* L. and curcumin [247,248]. Regulation of herbal products varies widely across jurisdictions, and the lack of standardized quality control for botanical preparations complicates dose–response assessments and comparisons across studies [249].

Several emerging strategies may bridge the translational gap. Humanized gnotobiotic mouse models (germ-free animals colonized with human fecal microbiota) offer better ecological validity for studying phytochemical–microbiome interactions under conditions closer to human physiology [250,251], while organoid and gut-on-a-chip technologies model human intestinal epithelium in contact with microbial communities, providing mechanistic data under conditions closer to human physiology than traditional cell lines [252]. Multi-omic approaches (metagenomics, metabolomics, transcriptomics) applied in well-stratified clinical studies are necessary for identifying response and non-response phenotypes and for discovering microbial metabolic pathways activated by plant compounds in the human gut [253,254].

To better understand the effects of phytomedicines on the microbiota, future studies should use metagenomics and/or culturomics to identify the bacterial species responsible for therapeutic effects, as current studies are usually limited to a single class of plant compounds; an analysis covering a wider range of chemical compositions would provide a more complete picture of how the gut microbiota processes phytomedicines [226].

Overall, recent research on the interaction between phytomedicines and gut microbiota in metabolic diseases is making promising progress. A deeper understanding of these interactions will clarify the therapeutic mechanisms of phytomedicines [226].

Preclinical research has accumulated robust mechanistic data, but translating microbiome-targeting herbal medicines into clinical practice requires improved bioavailability, standardized methodologies, consideration of microbiome heterogeneity, and evidence-based regulatory frameworks [255,256], while future clinical studies should be large-scale, well-controlled, and include personalized microbiome profiling, both as a stratification tool and as an outcome measure, in line with the principles of precision nutrition and microbiome-targeted medicine [257,258].

## 4. Microbiome-Targeted Interventions in Gastrointestinal Disorders

Microorganisms have been used in nutrition and medicine for millennia, long before their scientific identification [259,260]. Over the past two decades, the potential of prebiotics, probiotics, synbiotics, postbiotics, and fecal microbiota transplantation (FMT) [24,25] in the management of digestive diseases has been progressively uncovered, demonstrating effects on the regulation of the gut microbiome, improvement of epithelial integrity, and the exploitation of immunoregulatory mechanisms [261,262].

In this regard, prebiotics are non-viable substrates selectively utilized by resident microorganisms to confer a benefit to the host [263,264,265], while stimulation of *Lactobacillus* and *Bifidobacterium* species leads to increased production of short-chain fatty acids (SCFAs), with examples including inulin-type fructans (ITF), fructooligosaccharides (FOS), and galactooligosaccharides (GOS) [266,267]. Similarly, probiotics are live microorganisms with a genetically confirmed identity and demonstrate viability until the end of their shelf life, which confer health benefits to the host when administered in adequate amounts [268,269], including strains such as *Bifidobacterium animalis* subsp. *lactis* BB-12, *Lactobacillus rhamnosus* GG, and *Saccharomyces cerevisiae* var. *boulardii* [270,271].

Synbiotics are combinations of live microorganisms and substrates selectively used for growth [272], classified as complementary synbiotics and synergistic synbiotics [273,274], while postbiotics include non-viable microorganisms and/or their components, obtained through controlled inactivation processes, such as heat treatment or high pressure [275,276], with examples including heat-killed *Lactobacillus acidophilus*, *Bifidobacterium bifidum* MIMBb75, and pasteurized *Akkermansia muciniphila* [277,278]. In addition, FMT is a procedure involving the administration of fecal microbiota from a healthy human donor, with the aim of restoring microbial balance [279,280], and its mechanisms include increased microbial diversity, changes in microbial metabolites, altered intestinal permeability, and modulation of the gut–brain axis [281,282]. Furthermore, modifying the gut microbiota through probiotic and prebiotic fungi has been shown to reduce intestinal inflammatory burden, with implications for inflammatory bowel diseases and metabolic disorders, where a pro-inflammatory gut environment is associated with obesity and diabetes [266,283].

In this context, ex vivo experimental studies on inflamed mucosal tissue from patients with IBD have shown a decrease in pro-inflammatory cytokines and an increase in anti-inflammatory cytokines following administration of a nutraceutical compound consisting of *Hericium erinaceus*, berberine, quercetin, biotin, and niacin [282,284].

### Advanced Technological Innovations for the Microbiome Diagnosis of Gastrointestinal Disorders

**Figure 6:**
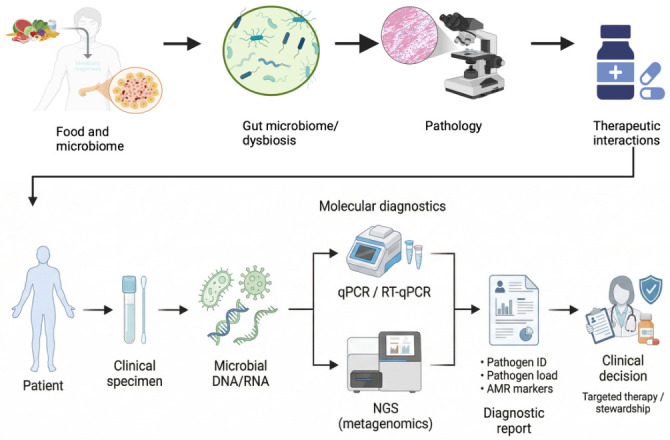
The interaction between plant-based foods, intestinal microbiome dysbiosis, pathological outcomes and the role of molecular diagnostics. Created in https://BioRender.com.

The diagnosis of infectious diseases of the gastrointestinal (GI) tract using traditional methods has been significantly transformed by molecular methods, which enable the rapid and accurate identification of pathogens and their genetic variants (Figure 6) [285].

Next-generation sequencing (NGS) has revolutionized comprehensive genotyping through its ability to sequence entire microbial genomes or cancer-associated mutations, offering a broad diagnostic scope in GI diseases and cancer, but at a higher cost and with greater technical complexity [285].

Shotgun metagenomics has become the dominant method, as it allows for the random sequencing of the entire genetic content of a microbiome [286,287], providing access to the study of all genes present in a sample and enabling both taxonomic and functional profiling [288,289], which is relevant in the context of identifying microbial responses to plant-derived bioactive compounds. However, although these methods provide extensive insights into microbial composition, they primarily assess the presence of organisms or genes without determining their active role within the microbiome [290,291].

To address this limitation, microbiome studies increasingly incorporate metatranscriptomics, which allows [292,293] for the analysis of actively expressed genes and the evaluation of microbial response to environmental changes over time [294,295]. Advances in metagenomic analysis, supported by next-generation sequencing, have significantly expanded the understanding of microbial communities, providing an advanced approach for identifying uncultivable microorganisms based on genetic information extracted directly from the environment [286,296].

Artificial intelligence and machine learning methods play an essential role in analyzing complex data from microbiomic studies, due to their multidimensional nature [297,298]. Machine learning (ML) is particularly useful in the study of gut microbiota, offering insights into how this microbial community influences health and disease, as well as enabling biomarker identification [299], such as fecal calprotectin and C-reactive protein (CRP) [300,301]. Furthermore, the integration of AI with large-scale metagenomic and multi-omics datasets has enhanced the understanding of microbiota functionality, allowing for the identification of functional genes, metabolic pathways, and potential biomarkers [302,303], which is relevant in the context of microbiome-mediated metabolism and response to bioactive compounds.

Despite these advances, the application of metagenomics, artificial intelligence, and molecular diagnostics in studies of plant-derived compounds remains limited by high interindividual variability, a lack of methodological standardization, and insufficient clinical validation. Furthermore, the correlation between microbiome profiles and phytochemical-influenced therapeutic outcomes has not yet been clearly established in human studies. These limitations highlight the need for integrative and standardized multi-omic frameworks to improve translational relevance [7].

## 5. Methods

The literature search was conducted between February and June 2026. A total of 587 articles were initially identified across PubMed/MEDLINE, Scopus, Web of Science, and ScienceDirect, using free-text terms and combinations with Boolean operators: “gut microbiome,” “dysbiosis,” “pharmacomicrobiomics,” “plant-based foods and compounds,” “drug-microbiome interactions,” “molecular pathways,” “diagnostic methods,” “microbiome modulation,” “bioactive metabolites,” “polyphenols and microbiota,” “flavonoids and gut microbiome,” “herbal medicine and microbiome,” and “animal model and human therapy and phytochemicals.” Duplicate publications were identified and removed before the full text was produced. Deduplication consisted of combining publications obtained from multiple databases and manually checking them by comparing titles, authors, and years of publication, which resulted in the removal of 89 duplicate entries and reduced the initial set of 587 to 498 unique publications. Full-text articles were then assessed for eligibility based on the following criteria: Inclusion: (1) original articles, systematic reviews, meta-analyses, and narrative reviews; (2) studies on microbiome-mediated interactions with plant foods, phytochemicals, or phytomedicines; (3) studies investigating the molecular mechanisms between phytochemicals, gut microbiota, and host signaling pathways; (4) studies with clinical or preclinical data on pharmacomicrobiomics, dysbiosis, and plant-based therapeutic strategies; (5) publications in English; Exclusion: (1) studies exclusively on synthetic drugs, without reference to plant compounds or interactions with the microbiome; (2) conference abstracts, editorials, and opinion pieces without original data.

After applying the inclusion/exclusion criteria, 307 references were retained. The selection prioritized studies published after 2020, with an emphasis on translational clinical data, molecular mechanisms, and studies linking results from animal models to therapeutic applications in humans.

## 6. Conclusions

The gut microbiota is considered one of the key elements influencing health through microbiome–host interactions across life stages, being associated with IBD, IBS, cardiovascular risk, allergies, and neurodegenerative diseases such as Alzheimer’s.

Within this context, plant-derived foods and medicines interact with the gut microbiome through microbial metabolism, generating bioactive metabolites that influence host signaling pathways and therapeutic response, highlighting the relevance of microbiome–phytochemical interactions in disease modulation.

Microbiome analysis can improve disease detection, monitoring, and personalized care. Advances in microbiome sequencing technologies are improving pathogen diagnosis and the understanding of microbiome functions, while meta-transcriptomics reveal active gene expression and microbial responses.

Future perspectives in microbiome research focus on integrating plant-derived compounds, multi-omics, artificial intelligence, and advanced diagnostics to improve personalized medicine and disease management. However, the clinical translation of microbiome–phytochemical interactions remains limited by interindividual variability and insufficient validation in human studies.

## Figures and Tables

**Table 2:** Plant-derived bioactive compounds, molecular targets and mechanisms of microbiome modulation.

Crt. No.	Representative Compounds and Sources	Dominant Bacterial Types	Molecular Target/ Mechanisms of Modulation
1.	Organic acids Malic acid - Immature hawthorns, *Malus malus* (L.) Voss (apples), and *Vitis* sp. (grapes). Mandelic acid - *Prunus amygdalus* Batsch (almonds), *Prunus armeniaca* L. (apricots), *Prunus mume* (Siebold) Siebold & Zucc. (peaches), *Vaccinium macrocarpon* Aiton (cranberries), *Rubus idaeus* L. (raspberries). Quinic acid - *Coffea* L. (coffee beans), fruits such as *Malus malus* (L.) Voss (apples), *Prunus mume* (Siebold) Siebold & Zucc. (peaches), *Actinidia chinensis* var. *deliciosa* (A. Chev.) (kiwi). Tartaric acid - *Citrus paradisi* Macfad. (grapes), *Vitis vinifera* L. (raisins and wine). Citric acid - *Citrus* L. (lemons, limes, and grapefruits).	*Acetobacter*, *Lactobacillus*, *Saccharomyces*, *Streptococcus*, *Enterococcus*, *Weissella*, *Lactococcus* *Bacillus aceticum*, *Lactobacillus*, *Saccharomyces cerevisiae*, *Escherichia*, *Shigella*, *Proteus*, *Paeniclostridium*, and *Streptococcus*	Malic acid—reducing the abundance of *Prevotella, Blautia*, *Prevotellaceae*_NK3B31 group, and *Collinsella* [208] Quinic acid could improve the dysbiosis of gut microbiota, inhibit intestinal inflammation by suppressing the activation of the MyD88/NF-κB signaling pathway in mice colon [212]
2.	Alkaloids Caffeine and Theophylline - *Coffea* L. (coffee beans), *Camellia sinensis* (L.) Kuntze (tea), *Theobroma cacao* L. (chocolate), certain soft drinks. Berberine - Roots, bark, and other structures of plants used in traditional Chinese/East Asian medicines such as *Coptis chinensis*, *Phellodendron amurense*, *Cercis chinensis*—Chinese Redbud, *Berberis aristata*, *B. petiolaris*, *B. vulgaris*, *B. aquifolium*, and *B. thunbergii*. Indoles - *Brassicaceae* Burnett (cruciferous vegetables).	*Bacteroides-Prevotella-Porphyromonas*, *Bifidobacterium*, *Clostridia cluster* XIV-a group, *Faecalibacterium prausnitzii* *Cryptobacterium curtum*, *Eggerthella* sp., and *Firmicutes*	Downregulation of the inflammatory cascade (COX-2, NF-κB, IL-6) [213] Regulation of DNA synthesis and induction of luminal detoxifying enzymes (β-glucuronidase, β-glucosidase, β-galactosidase, mucinase and nitroreductase) [214] Caffeine-related metabolites: theophylline, caffeine and paraxanthine; promoting the growth of SCFA-producing bacteria [215] Berberine can activate TLR4/MyD88/NF-κB signaling pathway to play an anti-inflammatory role [153]
3.	Organo-sulfur compounds Allicin, Alliin, Dihydroalliin - Vegetables such as *Allium sativum* L. (garlic) and *Allium cepa* L. (onions).	*Lactobacillus* *Bifidobacterium*	NK cell receptors such as TIGIT, NKG2D, KIR2DL3, and LFA-1 [216] A significant decrease in the percentage of T cells [216] Phytoncide exposure as well as boosts in the levels of effector molecules granzymes A/B, perforin, and granulysin [216]
4.	Polyphenols Anthocyanin - *Camellia sinensis* (tea); - Vegetables such as *Brassica oleracea* var. *italica* (broccoli), *Brassica oleracea* var. *capitata* f. *rubra* (red cabbage), *Daucus carota* L. (purple carrots); - Fruits such as *Prunus virginiana* L. (chokeberry), *Vaccinium praestans* Lamb. (Kamchatka berry), *Vaccinium myrtillus* L. (bilberry), *Vaccinium corymbosum* L. (blueberry), *Rubus occidentalis* L. (black raspberry), *Citrus paradisi* Macfad. (grapes) and *Oryza sativa* L. (black rice). Flavones Kaempferol: high levels in *Camellia sinensis* (L.) Kuntze (tea), *Citrus paradisi* Macfad. (grapes), *Brassica oleracea* var. *italica* (broccoli). Apigenin, Luteolin, Myricetin, Rutin, Sibelin, Quercetin. - *Malus malus* (L.) Voss (apple skins), *Brassica oleracea* var. *italica* (broccoli), *Apium graveolens* L (celery), ruit peels, *Vaccinium corymbosum* L. (bilberry), *V. oxycoccus* L. (cranberries), *Citrus paradisi* Macfad. (grapes), *Lactuca sativa* L. (lettuce), *Olea europaea* L. (olives), *Allium cepa* L. (onions). Flavanones - *Citrus* sp. (fruit and peel) [217].	*Bifidobacterium* spp. and *Lactobacillus* spp.	Anthocyanins—microbiome activity through SCFA-NLRP6, mTOR STAT3, and GRP43; decrease pH; decrease pathogenic bacteria [218] Molecular approach: blocking c- Jun, ERK1/2 [219]
5.	Glycosides Cardiac glycosides - Cardiac drug—digoxin Amygdalin (Amy) - Seeds of Rosaceae such as *Prunus amygdalus* Batsch (almonds) and *Prunus persica* (L.) Batsch (peach).	*Eggerthella lenta* *Enterobacter aerogenes*, *Streptococcus faecalis*, *Clostridium perfringens*	Digoxin inactivation by reduction; two-gene cardiac glycoside reductase (*cgr*) operon that was induced by digoxin in low-arginine conditions; dietary protein reduces the in vivo microbial metabolism of digoxin [209] Cyanogenesis of Amy; intestinal microbiota is involved in bidirectional regulation of toxicity and detoxification of Amy [220]
6.	Tetraterpenoid pigments Carotenoids (provitamin A carotenoids, lycopene, astaxanthin) - Orange, red, yellow and dark green fruits and vegetables, *Solanum* sp. L (tomatoes), *Cucurbita* sp. (pumpkins), *Mangifera indica* L. (mango), *Prunus armeniaca* L. (apricots), *Spinacia oleracea* L. (spinach), etc.	*Bifidobacterium adolescentis* and *Bifidobacterium longum* *Lactobacilli*	Enhancing or preserving intestinal barrier function; regulation of oxidative stress; stimulation of IgA [210]
7.	Tannins Hydrolysable tannins (Ellagitannins such as pedunculagin, vescalagin, castalagin, sanguiin and Gallotannins such as pentagalloyl-glucoside, hexagalloyl-glucoside, octagalloyl-glucoside) - Fruits *Punica granatum* L. (pommegranate)*, Fragaria ananassa* Duchesne (strawberry); - *Quercus* sp. L. (oak); - *Castanea sativa* Mill. (chestnut); - *Rhus semialata* Murray (sumac). Condensed tannins (epicatechin, catechin, procyanidin C1, C2, cinnamtannin A2) - *Clausena lansium* (Lour.) Skeels (bark); - *Camellia sinensis* (L.) Kuntze (tea); - *Vitis vinifera* L. (grapes); - *Persea americana* Mill. (avocado); - *Cinnamomum* sp. (cinnamon).	*Eubacterium cellulosolvens*, *Bacteroides distasonis*, *Bacteroides ovatus*, *Bacteroides uniformis*, *Enterococcus casseliflavus*, *Eubacterium ramulus*, Lachnospiraceae *CG191* the hydrolysis of tannins [211]	The metalloproteinases MMP-1, MMP-2 and MMP-9; inhibition of inducible NO synthase; increase CD4^+^ T-cell activation; TNF-α Urolithin A inhibited NF-κB and activator protein-1 (AP-1) activation as well as the phosphorylation of Akt and c-Jun N-terminal kinase (JNK); Urolithin A interfered with Akt/mTOR (mammalian target of rapamycin) [211] Anti-α-glucosidase, antityrosinase, antiproliferative and apoptotic activities [221]
8.	Essential oils Linalool, Limonene, Citral - *Aurantii dulcis aetheroleum* (orange essential oil); - *Limonis aetheroleum* (lemon essential oil).	*Lactobacillus*	Decreased of pro-inflammatory taxa, such as Desulfovibrionaceae (known sulfate-reducing pathobionts), Peptostreptococcaceae and Erysipelotrichaceae [219]

MyD88/NF-κB signaling—MyD88 (myeloid differentiation primary response 88); NF-κB—nuclear factor-κB; COX-2—Cyclooxygenase-2, prostaglandin-endoperoxide synthase 2; IL-6—interleukin 6, SCFA—Short-chain fatty acid; TLR4—toll-like receptor 4; NK—natural killer cell; TIGIT—T-cell immunoreceptor with Ig and ITIM domains; NKG2D—is an activating receptor (transmembrane protein), NKG2 family of C-type lectin-like receptors, KIR2DL3—Killer cell immunoglobulin-like receptor 2DL3 is a transmembrane glycoprotein expressed by the natural killer cells; LFA-1—Lymphocyte function-associated antigen 1; NLRP6—short for NOD-like receptor family pyrin domain containing 6; mTOR STAT3—a signaling axis where the mechanistic Target of Rapamycin (mTOR) acts as an activator of signal transducer and activator of transcription 3 (STAT3); GPR43—major host receptors for most SCFAs; c- Jun—Transcription factor Jun; ERK1/2—extracellular Signal-regulated kinases; MMP 1—Matrix metalloproteinase-1; MMP-2—Matrix metalloproteinase-2; MMP-9—Matrix metalloproteinase-9; NO—Nitric oxide; TNF-α—Tumor Necrosis Factor-alpha; Akt/mTOR—the serine/threonine kinase Akt, Akt at Ser473 by mTORC2 stimulates full enzymatic activity.

## Data Availability

Data is contained within the article.
